# Effect of COVID-19 on air pollution related illnesses in India

**DOI:** 10.1016/j.amsu.2022.103871

**Published:** 2022-05-26

**Authors:** Utkarsha Uday, Lakshmi Deepak Bethineedi, Muhammad Hasanain, Behram Khan Ghazi, Arsalan Nadeem, Prashastee Patel, Zaira Khalid

**Affiliations:** a West Bengal University of Health Sciences, Kolkata, India; b Andhra Medical College, Visakhapatnam, India; c Dow Medical College, Karachi, Pakistan; d Punjab Medical College, Faisalabad, Pakistan; e Allama Iqbal Medical College, Lahore, Pakistan; f Parul Institute of Medical Sciences and Research, Vadodara, India; g Karachi Medical and Dental College, Karachi, Pakistan

**Keywords:** Air pollution, Air quality index (AQI), Cardiovascular diseases, Occupational diseases,particulate matter (PM), Respiratory diseases, AQI, air quality index, NH_3_, ammonia, ADHD, attention deficit hyperactivity disorder, CO_2_, carbon dioxide, COPD, chronic obstructive pulmonary disease, COVID-19, coronavirus-19, μg/m³, micrograms per cubic metre, NCAP, national clean air programme, NO_2_, nitrogen dioxide, N95, non-oil 95, O_3_, ozone, PM, particulate matter, PPE, personal protective equipment, SLDBI, state level disease burden initiative, UNEP, united nations environment programme, USD, United States dollar, WHO, world health organization

## Abstract

Ambient air pollution level not only causes respiratory diseases but also cardiovascular diseases, besides, increased visits to the emergency department for asthma, chronic obstructive pulmonary disease (COPD), bronchitis, allergic rhinitis, attention deficit hyperactivity disorder (ADHD) in children and premature deaths in infants. The occurrence of Coronavirus-19 (COVID-19) pandemic is both, a boon and bane. Despite the deplorable situation aroused by the pandemic, strict lockdown measures implemented to curb the drastic spread of the disease, also culminated into astonishing outcomes that were not prioritized. This article illustrates the effects of the ongoing pandemic on air pollution and provides recommendations aimed at limiting it.

## 1 Introduction

India first reported a case of Coronavirus-19 (COVID-19) on 27 January 2020 [1]. As of 24 April 2022, a total of 43,057,545 cases have been reported in the nation, with 15,873 active cases [2]. The surge in COVID-19 cases forced many countries to implement strict public health policies such as social distancing and lockdown [3]. The lockdown response to COVID-19 has caused an unprecedented reduction in global economic and transport activity. Few studies have noticed significant changes in the air pollution levels during the lockdown periods [3]. The lockdown due to COVID-19 has led to decreased levels of Carbon dioxide (CO_2_) and Nitrogen dioxide (NO_2_) along with that of the particulate matter (PM) 2.5 levels across Europe, China and certain cities of India.

According to the World Health Organization (WHO), 91% of the world's population reside in places that exceed the WHO limits of poor air quality, resulting in 4.2 million deaths worldwide as a consequence of exposure to ambient air pollution [4]. In many developing nations, economic growths have exacerbated air pollutant emissions, with severe consequences for the environment and health. Poor air quality in Indian cities continues to present an ominous picture for health burdens attributable to ambient air pollution. Approximately, 38% of disease burden due to air pollution encompass cardiovascular diseases and diabetes in India, besides, pulmonary diseases. A study conducted by India's State Level Disease Burden Initiative (SLDBI) estimates that if the air pollution levels were lower than the minimum levels associated with health loss,the average life expectancy in India would have increased by 1.7 years [5].

The high burden of death and disease due to air pollution and its associated substantial adverse economic impact from loss of output could impede India's aspiration to be a $5 trillion economy by 2024. Successful reduction of air pollution in India strategically,would lead to substantial benefits for both the health of the population and the economy.

### 1.1 Air pollution and its associated diseases in India

Delhi, West Bengal, Madhya Pradesh, Bihar, Haryana, Gujarat and Punjab are the most air polluted states in India, according to the air quality index (AQI) and particulate matter (PM) 2.5 [6]. Agricultural exploitation such as crop burning in order to cater to the increasing population demands, coupled with the extensive automobile use, large-scale construction in urban areas, industrial pollutants and waste dumps are some of the major culprits behind air pollution across major cities in India, most notably Delhi [7]. These result in a slew of health difficulties, particularly affecting individuals with comorbidities. SA Rizwan et al. concluded that air pollution in Delhi has culminated into a myriad of diseases in different age groups [8]. A number of diseases have been associated with inhalation exposure to airborne PM: respiratory disorders whose effects range from minor symptoms such as coughs and dyspnea to severe ones such as acute respiratory infections, asthma, and pneumonia, chronic obstructive lung diseases such as bronchitis, cardiovascular disease, tuberculosis, lung cancer, resulting in frequent emergency room visits [9]. In addition, allergic rhinitis, ocular irritation, cataract, blindness, besides, perinatal effects such as stillbirths and low birth weights are also associated with air pollution [10]. However, the health end point that is most clearly defined is death, and many epidemiological studies in developed countries focus on obtaining relationships between mortality rates and ambient levels of pollution [10]. Few cases of attention deficit hyperactivity disorder (ADHD) in children have also been observed [11].

High levels of chronic morbidity exert their own toll and pose severe strains on the health care infrastructure. For an instance, increased incidence of pulmonary diseases will lead to increased sale of drugs such as cetrizine (an antihistamine), salbutamol (a bronchial dilator), bromhexine (mucolytic), amoxicillin, and erythromycin (antimicrobials), eventually adding to higher antibiotic resistance or even an emergence of a more resistant case.This increases the risk of transmission many folds in overcrowded dwellings and slums. Moreover, the high costs of sophisticated pharmacological drugs and devices such as metered dose inhaler or a nebulizer makes affordability tougher for the lower economic sections. Also, tobacco smoking, occupational exposures to air pollution, health status, and co-pollutants can be significant confounders in any epidemiological study involving pulmonary disease.

A significant fraction of population work in manufacturing and production industries such as coal, iron and steel, asbestos as well as small-scale operations like welding and metal plating, where they are exposed to high levels of PM. In urban India, typically the workforce in such sectors lives close to the work place and is exposed to ambient emissions from these industries as well. Occupational exposures to a wide variety of airborne dusts, gases, and fumes such as grain dust, wood dust, and various metal fumes like nickel, chromium, and cobalt add to the disease burden such as urinary bladder carcinoma and are not limited to respiratory and cardiovascular diseases only. Some occupational diseases attributed to ambient exposures are pneumoconiosis (from coal dust), pulmonary fibrosis (from silica or asbestos exposures), and lung cancer. Recently, the WHO has lowered the threshold for acceptable pollution levels with the issuance of more strict air quality criteria for areas where human health can be considered safe. While a concentration of PM 2.5 or 25 micrograms per cubic metre (μg/m³) was previously considered safe, the new standards have reduced the allowed levels to 15 μg/m³ [12].

### 1.2 Effects of COVID-19 on air pollution levels in India

A global decrease in CO_2_ emissions to 8.8% was observed in the first half of 2020 [13]. Giani et al. reported a dip in the PM 2.5by 2·2 μg/m³ across Europe, whereas a decrease of 14·5 μg/m³ across China had been observed [14]. Similarly, a cumulative reduction of PM 2.5, PM 10, Sulfur dioxide (SO_2_) and Ammonia (NH_3_) by 49–73%, 17–63%, 15–58%, and 30–74%, respectively in the air quality across Delhi, Mumbai, Kolkata, and Chennai, has been reported by Pant et al. [15]. Another study done on 6 mega cities of India namely, Delhi, Lucknow, Kolkata, Mumbai, Chennai and Jaipur, reported an average final reduction of 37.42% in AQI PM 2.5 and 65.80% in AQI NO_2_ in each of these cities [16].

The significant reduction in air pollution during the COVID-19 lockdowns can be attributed to decreased anthropogenic activities and favorable meteorology. Reportedly, temporary suspension of vehicular traffic in South Coast Air Basin, USA, led to a reduction in PM 2.5 by 18–36% [17]. Industrial emissions in Kolkata and Chennai, vehicular exhaust and dust emissions in Delhi, Hyderabad, and Mumbai are major contributors to PM 2.5 in India [16,18]. Both exhaust and non-exhaust vehicular emissions make up about 60% of the PM 2.5 load in Delhi [19]. In Hyderabad, the vehicular exhaust (31%) and re-suspended dust (26%) contribute significantly to PM 2.5, followed by combustion (9%), industries (7%) and burning (6%) [20].

Secondly, increased air pollution is associated with adverse health outcomes. About 8.8 million people die annually due to poor air quality adding to the cost burden of over United States dollar (USD) 1–3 trillion [21]. Ambient air pollution is the leading cause of mortality due to environmental risk factors, and the fifth overall, preceding tobacco smoking and human immunodeficiency virus (HIV) or acquired immunodeficiency syndrome (AIDS) [21]. Similarly, another study reported that regular exposure to PM 2.5 and Ozone (O_3_) led to greater risk for premature deaths in adults (≥25 years) from cardiovascular diseases (CVD), respiratory diseases such as obstructive lung diseases, lung cancer and stroke [22]. Interestingly, 60% deaths stem from CVD as a direct causation of air pollution [21]. The probability of acquiring air pollution related diseases is directly proportional to the annual PM 2.5 concentration the population is exposed to [14]. Moreover, COVID-19 is also found to be positively correlated with air pollution. Zhue et al. observed that a 1 μg/m3 elevation in PM 2.5 concentration, led to an increase of 0.22% in COVID-19 cases in China [23]. Another study reported an increase of 13.0–21.4% COVID-19 mortality rate with an elevation of 1 μg/m3 in PM 2.5 concentration in the Netherlands [22]. Similarly, Indian cities with improved air quality reported lower deaths due to COVID-19 as compared to those with poor air quality [24].

Thirdly, it is estimated that the 2021 lockdown related reduction in PM 2.5 levels, prevented approximately 2190 deaths across Europe while, 24,200 deaths across that of China [21]. The study also predicts that the continuation of strict lockdowns throughout 2021 could have averted more than 300,000 deaths due to air pollution [21].

## 2 Recommendations

Though lockdowns have been effective at improving air quality, they are not feasible due to economic repercussions. Hence, it is essential that the policy makers adopt long term alternatives using prior data to curb air pollution. Worldwide fossil fuel burning makes up 50%–60% of artificial airborne pollutants [25]. It is estimated that if fossil fuel combustion is discontinued, the global life expectancy could increase by 1.1 years [26]. A great way to control air pollution is by taking measures to reduce vehicular emissions. Using high quality gasoline and catalytic converters would be beneficial. Public transport with greater fuel efficiency, should be made easily available to the masses and use of private vehicles should be discouraged. Public bike sharing (PBS) system similar to China should be implemented, which is not only eco-friendly but also beneficial for health. Institutions such as offices should promote and incentivize car-pooling. Work from home should be given preference to, whenever feasible. Lockdown measures have also beneficially reduced the global energy demand, resulting in the reduction of greenhouse gas emission. Exploring the use of renewable energy sources that would replace the dependency on fossil fuels should be emphasized upon.

Since the outbreak of ongoing pandemic, medical waste generation, such as needles and syringes, have increased globally, besides, the production and use of plastic based personal protective equipment (PPE) kits.Non-oil 95 (N-95) masks, made of polypropylene and Tyvek, a potential source of micro-plastic fibers used to make protective suits, have the ability to persist in the environment for a longer time. These release dioxin and other toxic elements into the environment [27]. Being a public health hazard, which is detrimental to the environment, it is imperative to undertake proper waste management in accordance with WHO guidelines [28]. Haphazard waste disposal of household scraps and dumping in open places should be avoided. The masses need to be educated about infectious diseases, waste segregation, management and handling through mass media campaigns and door to door community-based programs supported by all the stakeholders.

Steps must be taken to ensure that industrial and municipal wastes are recycled properly [29]. The possibility of an artificial sequestration of carbon captured from emissions produced by gasification plants followed by their storage underground in coal seams can be explored.A carbon tax may be imposed based on the magnitude of CO_2_ emissions to encourage conservation.

Small individual efforts such as turning off lights when not in use, avoiding the use of air-conditioners and plastic bags, and planting more trees, would most definitely make an impact in the long term. Additionally, ecotourism should be promoted, keeping in mind the social and hygienic practices so as to consolidate cultural preservation and biodiversity conservation whilst supporting livelihoods [30]. With city action plans having been recently prepared and the national clean air programme (NCAP) setting goals to improve the air quality by 2024, this is indeed an appropriate time to conduct analyses to impact and bend the curve of air pollution substantially. Collective international efforts to achieve the sustainable environmental goals through protection of global environmental resources, global climate and biological diversity, must be encouraged [31]. The United Nations Environment Programme (UNEP) can play an important hand in aiding the cause by implementing timely policies and conducting international seminars, conventions - enhancing coordination amidst global leaders for collective action towards realizing an eco-friendly environment.

## 3 Conclusion

It has been established that ambient air pollution levels cause or exacerbate several diseases in the general population. However, lockdown measures due to COVID-19, have resulted in a decrease in the pollutant levels including CO_2_ and NO_2_ along with that of PM 2.5 levels. Despite a reduction in mortality as well as morbidity due to air pollution, lockdown is not a practical solution due to its detrimental effects on the economy, job recession, loss of livelihood of migrant workers among many others. Hence a more pragmatic long-term plan for sustainable management of the environment should be devised with an emphasis on proper waste management and the use of eco-friendly energy resources.

## Funding

This research did not receive any specific grant from funding agencies in the public, commercial, or not-for-profit sectors.

## Ethical approval

Research studies involving patients require ethical approval. Please state whether approval has been given, name the relevant ethics committee and the state the reference number for their judgement.

## Consent

NA.

## Author contribution

All the authors contributed equally for study concept or design, data collection, data analysis or interpretation and writing the paper.

## Registration of research studies

NA.

1. Name of the registry:

2. Unique Identifying number or registration ID:

3. Hyperlink to your specific registration (must be publicly accessible and will be checked):

## Guarantor

Utkarsha Uday.

## Declaration of competing interest

The authors declare that they have no known competing financial interests or personal relationships that could have appeared to influence the work reported in this paper. All authors should have made substantial contributions to all of the following: (1) the conception and design of the study, or acquisition of data, or analysis and interpretation of data, (2) drafting the article or revising it critically for important intellectual content, (3) final approval of the version to be submitted.

## References

[1] (2020 May). Indian J. Med. Res..

[2] COVID-19 portal. Government of India. Available at: https://www.mygov.in/covid-19. Accessed on: 24th April, 2022.

[3] Ju M.J., Oh J., Choi Y.H. (2021 Jan 1). Changes in air pollution levels after COVID-19 outbreak in Korea. Sci. Total Environ..

[4] Air Pollution. World Health Organization. Published: 22^nd^ September, 2021. Accessed on: 10^th^ December, 2021. Available at: https://www.who.int/health-topics/air-pollution#tab=tab_1.

[5] (2019). The impact of air pollution on deaths, disease burden, and life expectancy across the states of India: the Global Burden of Disease Study 2017. Lancet Planet. Health.

[6] India air quality index (AQI) and air pollution information. Air Visual. (n.d.). Available at: https://www.iqair.com/in-en/india. Accessed on: September 24, 2021.

[7] Top 8 Main Causes for Air Pollution in Delhi. The Times of India. Published: 15^th^ November, 2017. Accessed on: 10^th^ December, 2021. Available at: https://timesofindia.indiatimes.com/life-style/health-fitness/health-news/top-8-main-causes-for-air-pollution-in-delhi/articleshow/61626744.cms.

[8] Rizwan S., Nongkynrih B., Gupta S.K. (2013). Air pollution in Delhi: its magnitude and effects on health. Indian J. Community Med. : off publ Indian Assoc Prevent Soc Med.

[9] Mannucci P.M., Harari S., Franchini M. (2019 Dec). Novel evidence for a greater burden of ambient air pollution on cardiovascular disease. Haematologica.

[10] Rajak R., Chattopadhyay A. (2020 Nov 1). Short and long term exposure to ambient air pollution and impact on health in India: a systematic review. Int. J. Environ. Health Res..

[11] Myhre O., Låg M., Villanger G.D., Oftedal B., Øvrevik J., Holme J.A., Aase H., Paulsen R.E., Bal-Price A., Dirven H. (2018 Sep 1). Early life exposure to air pollution particulate matter (PM) as risk factor for attention deficit/hyperactivity disorder (ADHD): need for novel strategies for mechanisms and causalities. Toxicol. Appl. Pharmacol..

[12] WHO Global Air Quality Guidelines: Particulate Matter (PM2.5 and PM10), Ozone, Nitrogen Dioxide, Sulfur Dioxide and Carbon Monoxide. World Health Organization (WHO). Published on: September 22, 2021. Accessed on: 10^th^ December, 2021. Available at: https://www.who.int/publications/i/item/9789240034228. 34662007

[13] Liu Z., Ciais P., Deng Z. (2020). Near-real-time monitoring of global CO_2_ emissions reveals the effects of the COVID-19 pandemic. Nat. Commun..

[14] Giani P., Castruccio S., Anav A., Howard D., Hu W., Crippa P. (2020). Short-term and long-term health impacts of air pollution reductions from COVID-19 lockdowns in China and Europe: a modelling study. Lancet Planet. Health.

[15] Pant G., Alka, Garlapati D., Gaur A., Hossain K., Singh S.V., Gupta A.K. (2020 Dec). Air quality assessment among populous sites of major metropolitan cities in India during COVID-19 pandemic confinement. Environ. Sci. Pollut. Res. Int..

[16] Agarwal A., Kaushik A., Kumar S., Mishra R.K. (2020 Jul 23). Comparative study on air quality status in Indian and Chinese cities before and during the COVID-19 lockdown period. Air Qual Atmos Health.

[17] Quiros D.C., Zhang Q., Choi W., He M., Paulson S.E., Winer A.M., Wang R., Zhu Y. (2013). Air quality impacts of a scheduled 36-h closure of a major highway. Atmos. Environ..

[18] Ravindra K., Singh T., Biswal A., Singh V., Mor S. (2021 May). Impact of COVID-19 lockdown on ambient air quality in megacities of India and implication for air pollution control strategies. Environ. Sci. Pollut. Res. Int..

[19] Sahu S.K., Beig G., Parkhi N.S. (2011). Emissions inventory of an thropo-genic PM2. 5 and PM10 in Delhi during commonwealth games 2010. Atmos. Environ..

[20] Gummeneni S., Yusup Y.B., Chavali M., Samadi S.Z. (2011). Source apportionment of particulate matter in the ambient air of Hyderabad city, India. Atmos. Res..

[21] Wolhuter K., Arora M., Kovacic J.C. (2021 Sep). Air pollution and cardiovascular disease: can the Australian bushfires and global COVID-19 pandemic of 2020 convince us to change our ways?. Bioessays.

[22] JyotiMaji Kamal, Namdeo Anil, Bell Margaret, Goodman Paul, Shiva Nagendra S.M., Barnes Joanna H., De Vito Laura, Hayes Enda, Longhurst James W., Kumar Rakesh, Sharma Niraj, Sudheer Kumar Kuppili&DheerajAlshetty (2021). Unprecedented reduction in air pollution and corresponding short-term premature mortality associated with COVID-19 lockdown in Delhi, India. J. Air Waste Manag. Assoc..

[23] Zhu Y., Xie J., Huang F., Cao L. (2020). Association between short-term exposure to air pollution and COVID-19 infection: evidence from China. Sci. Total Environ..

[24] Naqvi H.R., Datta M., Mutreja G., Siddiqui M.A., Naqvi D.F., Naqvi A.R. (2021 Jan 1). Improved air quality and associated mortalities in India under COVID-19 lockdown. Environ. Pollut..

[25] Pozzer A., Dominici F., Haines A., Witt C., Münzel T., Lelieveld J. (2020). Regional and global contributions of air pollution to risk of death from COVID-19. Cardiovasc. Res..

[26] Lelieveld J., Pozzer A., Pöschl U., Fnais M., Haines A., Münzel T. (2020). Loss of life expectancy from air pollution compared to other risk factors: a worldwide perspective [published correction appears in Cardiovasc Res. 2020 Jun 1;116(7):1334]. Cardiovasc. Res..

[27] Ellabban O., Abu-Rub H., Blaabjerg F. (2014). Renewable energy resources: current status, future prospects and their enabling technology. Renew. Sustain. Energy Rev..

[28] Global analysis of health care waste in the context of COVID-19. World Health Org(WHO). Published on: 1stFebruary, 2022. Accessed on: 1stFebruary, 2022. Available at: https://www.who.int/publications/i/item/9789240039612.

[29] Hysa E., Kruja A., Rehman N.U., Laurenti R. (2020). Circular economy innovation and environmental sustainability impact on economic growth: an integrated model for sustainable development. Sustainability.

[30] Islam S.M.D., Bhuiyan M.A.H. (2018). Sundarbans mangrove forest of Bangladesh: causes of degradation and sustainable management options. Environ. Sustain..

[31] ICIMOD . International Centre for Integrated Mountain Development (2020). COVID-19 impact and policy responses in the hindu kush himalaya. https://lib.icimod.org/record/34863.

